# Supplementary figures and images for: Plasma extracellular vesicle synaptic proteins as biomarkers of clinical progression in patients with Parkinson’s disease
Source: eLife. 2024 Mar 14;12:RP87501. doi: 10.7554/eLife.87501 (PMC10939498; doi:10.7554/eLife.87501)

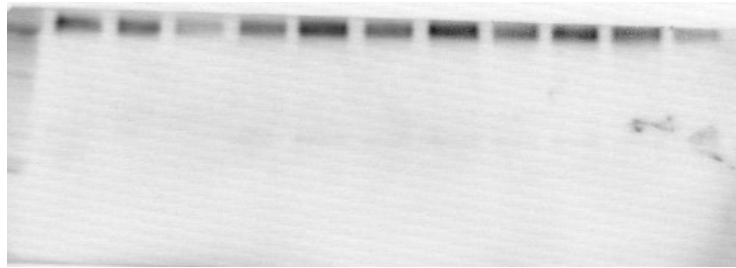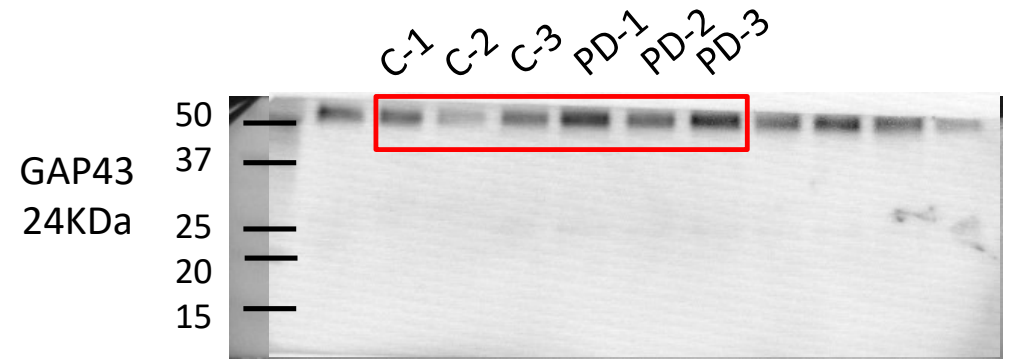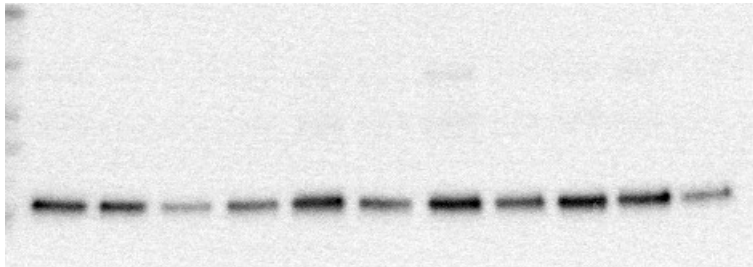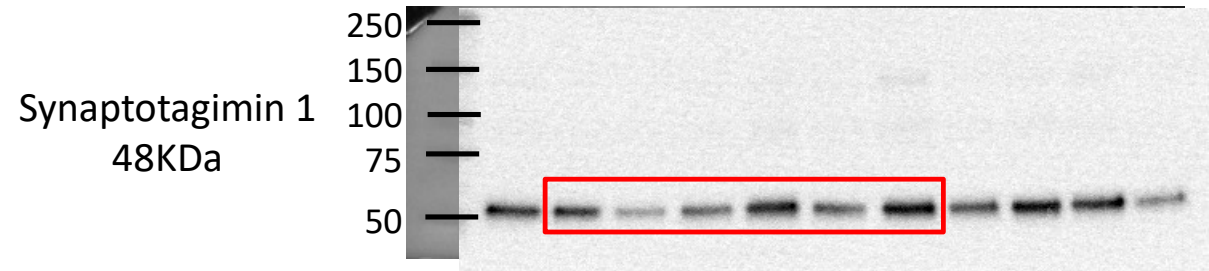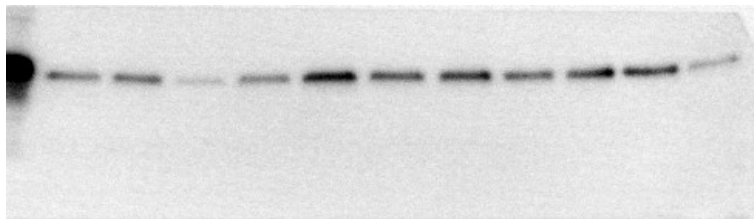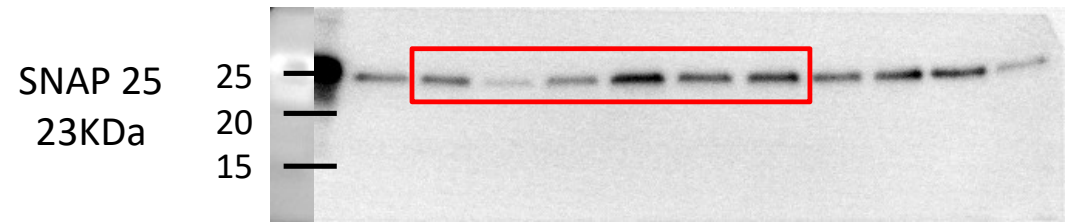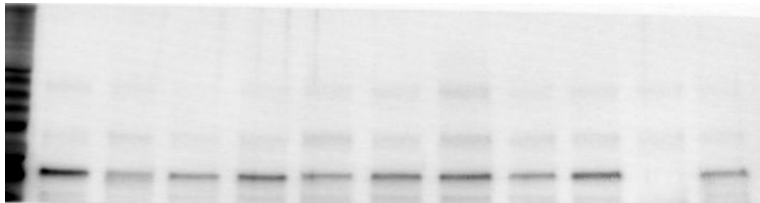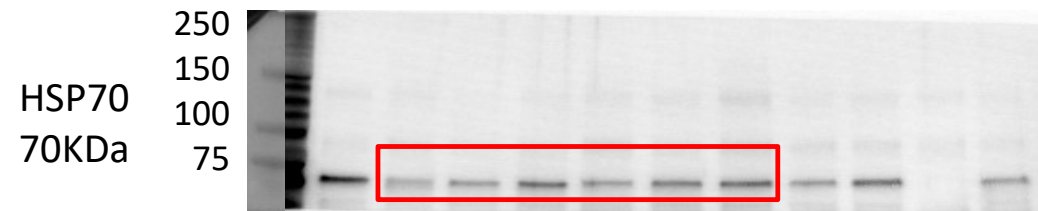

Supplement: Figure 1—source data 1. [file elife-87501-fig1-data1.pdf]
