# Supplementary material for: Plasma extracellular vesicle synaptic proteins as biomarkers of clinical progression in patients with Parkinson’s disease
Source: eLife. 2024 Mar 14;12:RP87501. doi: 10.7554/eLife.87501 (PMC10939498; doi:10.7554/eLife.87501)
Supplement: Supplementary file 1. — UPDRS, Unified Parkinson’s Disease Rating Scale; AR, akinetic rigidity; PIGD, postural instability and gait disturbance; MMSE, Mini-Mental Status Examination; MoCA, Montreal Cognitive Assessment. [file elife-87501-supp1.docx]

Supplementary File 1

Association between the baseline plasma EV synaptic proteins with the clinical severity in people with Parkinson’s disease at follow-up with the adjustment of age, sex, disease duration and the baseline severity of corresponding item, presented as standardized B and p value.

|  | UPDRSII | UPDRSIII |  |  |  | MMSE | MoCA |
| --- | --- | --- | --- | --- | --- | --- | --- |
|  |  |  | Tremor | AR | PIGD |  |  |
| SNAP-25 | 0.137 (0.132) | 0.126 (0.135) | -0.032 (0.753) | 0.090 (0.278) | **0.216 (0.004)** | -0.069 (0.400) | 0.014 (0.811) |
| GAP-43 | 0.127 (0.162) | 0.111 (0.186) | -0.034 (0.737) | 0.084 (0.312) | **0.166(0.030)** | -0.041 (0.617) | -0.010 (0.862) |
| Synaptomagtin-1 | 0.108 (0.223) | 0.064 (0.41) | -0.055 (0.582) | 0.042 (0.609) | 0.139 (0.06) | -0.009 (0.911) | -0.002 (0.967) |

UPDRS, unified Parkinson Disease rating scale; AR, akinetic rigidity; PIGD, postural instability and gait disturbance; MMSE, mini-mental status examination; MoCA, Montreal cognitive assessment.
